# A preoperative prediction model for ipsilateral axillary lymph node metastasis of breast cancer based on clinicopathological and ultrasonography features: a prospective cohort study

**DOI:** 10.3389/fonc.2025.1536984

**Published:** 2025-10-01

**Authors:** Xinyi Guo, Yue Ling, Yulan Peng, Qiuwen Tan, Yanyan Xie, Haina Zhao, Qing Lv

**Affiliations:** ^1^ Department of General Surgery, West China Hospital, Sichuan University, Chengdu, China; ^2^ Breast Disease Center, West China Hospital, Sichuan University, Chengdu, China; ^3^ Department of Ultrasound, West China Hospital, Sichuan University, Chengdu, China

**Keywords:** breast cancer, axillary lymph nodes (ALN), ultrasonography (US), nomogram, preoperative

## Abstract

**Background:**

For breast cancer, developing non-invasive methods to accurately predict axillary lymph node (ALN) status before surgery has become a general trend. This study aimed to develop and evaluate a nomogram to predict the probability of ALN metastasis (ALNM) preoperatively based on clinicopathological and ultrasonography (US) features.

**Methods:**

Patients diagnosed with breast cancer by preoperative histopathologic biopsy in West China Hospital from 1 August, 2022 to 31 January, 2024 and undergoing surgical treatment with preoperative US in West China Hospital were prospectively included. Preoperative clinicopathological and US features, along with postoperative pathological ALN status, were collected. Patients included were randomly divided into a training set and a test set (7:3). In the training cohort, the independent predictors of ALNM were obtained by univariate and multivariate binary logistic regression analyses and were used to develop a binary logistic regression model presented as a nomogram. Model performance was evaluated by receiver operating characteristic (ROC) curve, calibration curve, and decision curve analysis (DCA).

**Results:**

A total of 610 patients were included for analysis: 427 in the training set and 183 in the test set. Molecular subtypes, tumor infiltration of the subcutaneous layer, tumor infiltration of the retromammary space, lymph node (LN) short axis, LN long/short (L/S) axis ratio, LN corticomedullary demarcation, and LN cortical thickness evenness were independent predictors of ALNM. The nomogram showed good discrimination with an area under the ROC curve (AUC) of 0.854 for the training set and 0.822 for the test set, presented good agreement between predicted and observed probabilities, and acquired net benefit across a wide threshold range.

**Conclusions:**

The nomogram demonstrated strong discrimination, calibration, and clinical net benefit to assist clinical decisions.

## Introduction

Breast cancer is the most common malignancy and the leading cause of cancer-related deaths in women (1). In, 2022, over 2 million new cases and 660,000 deaths occurred globally, with age-standardized incidence and mortality rates of 46.8 and 12.7 per 100,000, respectively (1). Metastasis is a major factor in cancer mortality (2), and lymph node (LN) status is crucial for breast cancer prognosis (3–5). For breast cancer, axillary lymph node (ALN) status is essential for staging, treatment, and prognosis (6–8), with ALN metastasis (ALNM) considered an indicator of recurrence and survival rates (9).

ALN dissection (ALND) was once the gold standard for ALNM assessment but was replaced by sentinel lymph node biopsy (SLNB) (10) due to serious complications, such as pain, restricted shoulder movement, lymphedema, paresthesia, and numbness (11–14), marking a milestone in surgical de-escalation (15). Although SLNB is less invasive, it still poses the abovementioned moderate risks (16). Notably, 75% of patients with negative preoperative ALN ultrasonography (US) (17) and approximately 40% with positive US (18) had negative ALN upon pathological examination. The Sentinel Node vs Observation After Axillary Ultrasound (SOUND) randomized clinical trial showed that omitting axillary surgery was non-inferior to SLNB in patients with tumors ≤2 cm and negative ALN US (15), forecasting another shift in surgical de-escalation. Accurately identifying ALN status preoperatively is essential to avoid unnecessary axillary surgery in patients without ALNM.

US-guided core needle biopsy (CNB) and fine-needle aspiration (FNA) offer certain sensitivity, excellent specificity, and good positive predictive value (PPV) to preoperative ALN status assessment (19–24). To protect important vessels and nerves close to ALN, FNA is more commonly used but still carries potential risks (25) and has an unsatisfactory false-negative rate (FNR) of up to nearly 30% (24, 26). To avoid unnecessary invasive biopsies, developing non-invasive methods to accurately predict ALN status before surgery has become a general trend (27).

Currently, non-invasive imaging examinations such as US, mammography (MG), magnetic resonance imaging (MRI), and positron emission tomography–computed tomography (PET–CT) are used to predict ALN status preoperatively (27). US is preferred due to its convenience, low cost, no ionizing radiation, and abundant morphological information (28–30). Studies have indicated that LNs with asymmetry, thickened or irregular cortex, round shape and roundness index of approximately 1, enlarged size, ill-defined margins, or disappeared fatty hilum were more prone to metastatic LNs (10, 31, 32). However, there are no official consensus criteria to classify benign versus metastatic LNs based on US (10).

Previous studies created models to predict ALNM based on US features, but insufficiencies still existed (27, 29, 32–34). First, the features incorporated were inadequate, only involving LNs (29, 32, 34) or tumors (27), without important features like tumor infiltration of the subcutaneous layer or retromammary space (27, 29, 32–34). Second, these models relied on clinicopathological features from surgical specimens, unsuitable for preoperative assessment (33). Third, some models lacked comprehensive evaluation (27, 29, 32, 33). Until now, few studies have developed a comprehensive model based on preoperative clinicopathological and US features to predict ALNM, with adequate evaluation.

More comprehensively combining preoperative clinicopathological and US features by constructing a quantifiable model to more accurately predict preoperative ALN status needs urgent exploration. This study aimed to explore the risk factors of ALNM prospectively from preoperative clinicopathological features, as well as US features of tumor and LNs, and develop a nomogram to predict ALNM probability preoperatively for breast cancer, with a more comprehensive evaluation.

## Materials and methods

### Patient selection

Patients diagnosed with breast cancer by preoperative histopathologic biopsy in West China Hospital, Sichuan University, from 1 August, 2022 to 31 January, 2024 were prospectively included. The pathological diagnosis was based on the World Health Organization (WHO) Classification of Breast Tumors (35). Patients meeting the following criteria were excluded: 1) cancer primary focus and/or LN metastasis focus has been resected via breast surgery (including breast-conserving surgery, mastectomy, and even Mammotome) and/or axillary surgery (including ALND and SLNB), 2) inflammatory breast cancer, 3) a history of malignancy, and 4) necessary clinicopathological data were absent.

The study was conducted in accordance with the Declaration of Helsinki and approved by the Ethics Committee of West China Hospital, Sichuan University. Written informed consent was obtained from all patients.

### Clinicopathological feature collection

Patients’ clinical information was mainly collected by face-to-face inquiry, and histopathologic information of tumors was obtained from the electronic medical records. Clinical features included age at diagnosis, body mass index (BMI), age at menarche, the number of pregnancies and deliveries, menopause status at diagnosis, and neoadjuvant therapy (NAT) or not. BMI was calculated by formula weight/height^2^ (kg/m^2^), and the height and weight were measured in an outpatient setting. The histopathologic reports of preoperative US-guided CNB for breast primary tumors, including estrogen receptor (ER), progesterone receptor (PR), human epidermal growth factor receptor 2 (HER2), Ki-67, and histologic types, were recorded. The features of the largest tumor were recorded for patients with multiple tumors. The status of ER, PR, HER2, and Ki-67 was evaluated by immunohistochemical (IHC) staining, and HER2 needed extra detection by fluorescence *in situ* hybridization (FISH) when IHC showed 2+. ER or PR was identified as a positive result if the positivity rate was ≥1%, and HER2 was identified as a positive result if IHC staining presented 3+, or IHC showed 2+ but FISH showed positive. According to the St Gallen International Expert Consensus, 2013 (36), based on the status of ER, PR, HER2, and Ki-67, breast cancer was categorized into four molecular subtypes, as follows: luminal A subtype (ER+, PR ≥ 20%, HER2−, Ki-67 < 14%), luminal B subtype (ER+/PR+ but did not meet the condition of luminal A), HER2-enriched subtype (ER−, PR−, HER2+), and triple-negative subtype (ER−, PR−, HER2−).

### US data collection

The US scans were evaluated 3–5 days before surgery by two ultrasound experts (with 5–10 years of experience in breast US) blinded to the axillary surgery plan, and the images and features for breasts and ALN were collected. They reached a consensus through discussion when any disagreements in the independent analysis were encountered. US features of the tumor included tumor size (maximum diameter of breast lesion), location, distance to the nipple, blood flow signals, infiltration status of the subcutaneous layer, and retromammary space. In this study, the blood flow signals of tumors were divided into two categories based on Adler grades: poor (Adler grades 0–1) and abundant (Adler grades 2–3) blood flow signals (37). The characteristics of the largest tumor were recorded for patients with multiple tumors. In addition, US features of LNs contained long and short axes, blood flow signal types and grades, margin, corticomedullary demarcation, and cortical thickness and evenness. The cortical thickness was measured based on the thickest location. Then, the LN long/short (L/S) axis ratio by long and short axes was calculated. The characteristics of the most suspicious LN (the largest one usually) were recorded.

### Pathological ALN status

The surgery was performed by six specialists in breast surgery with more than 10 years of experience in breast cancer surgery. The ALN status reported by the postoperative histopathologic examination results of ALND or SLNB was recorded. Then, patients were divided into ALNM and non-ALNM groups by the ALN status. Macro-metastases (>2 mm) and micro-metastases (0.2–2 mm) were identified as ALNM, while isolated tumor cells (ITCs) (<0.2 mm) and negative SLN were considered non-ALNM (38).

### Follow-up and research management

All the patients were followed up. The end-point of follow-up was the acquirement of ALN status by postoperative histopathologic examination. During the follow-up, NAT status was checked via electronic medical records when the patients were admitted to the hospital for surgery. The patients who were not undergoing a surgical treatment or refused preoperative US in West China Hospital were lost to follow-up.

The patients’ data were registered in an Excel spreadsheet and were managed by a dedicated researcher. Clinicopathological feature collection was performed by two researchers in charge of acquisition and recording. The US features were recorded by one of the two ultrasound experts after a consensus was reached. Another two researchers were responsible for follow-up until postoperative pathological ALN status was recorded.

### Statistical analysis

Statistical analysis was performed using SPSS 23.0 and the R software ver.4.3.2. Continuous variables were expressed as mean ± standard deviation (SD), while categorical variables were presented as numbers and percentages of the group they belong to and analyzed by χ^2^ test (Yates’ correction if necessary) or Fisher’s exact test, which was used to compare the data distribution of the training and test cohorts.

The patients included were randomly divided into a training set and a test set according to the proportion of 7:3. In the training cohort, the variables associated with ALN status were preliminarily screened by univariate binary logistic regression analysis. Those significant variables in univariate analysis were included in multivariate binary logistic regression analysis, using the forward stepwise (likelihood ratio), to acquire the independent predictors of ALNM. The p-values, odds ratios (ORs), and 95% confidence intervals (CIs) were reported to present the analysis. The statistical analyses were two-sided, and p < 0.05 was considered statistically significant. The collinearity diagnosis of independent predictors was performed, and then the tolerance and variance inflation factor (VIF) were calculated.

The binary logistic regression model to predict ALNM was constructed using independent predictors and presented as a nomogram. The Hosmer–Lemeshow goodness-of-fit test was used to evaluate the fit level of the model. For the training and test cohorts, the discrimination of the model was evaluated by the receiver operating characteristic (ROC) curve to calculate the area under the ROC curve (AUC). Next, for both cohorts, the calibration of the model was evaluated by bootstrapping with 500 resamples and presented by the calibration curve. Last, to evaluate the clinical value of the nomogram, the net benefits of the model for both cohorts were measured using decision curve analysis (DCA) and shown by the DCA curve. Moreover, sensitivity, specificity, accuracy, PPV, negative predictive value (NPV), detection rate, and FNR of the model at different threshold values in the training and test sets were calculated to provide references for threshold value selection in situations with different requirements.

## Results

### Patients’ characteristics

**Figure 1:**
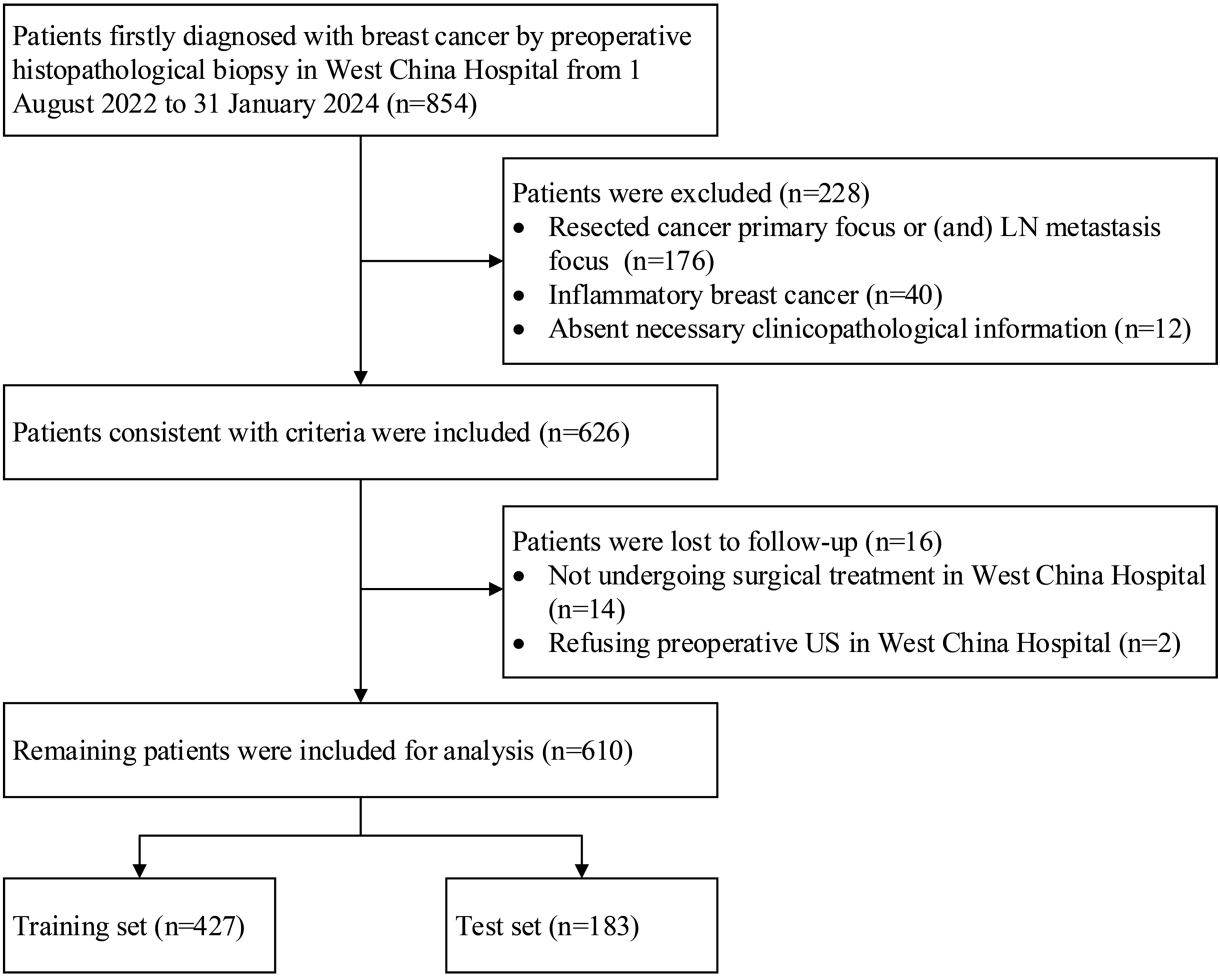
Flowchart of patients who were included, excluded, and lost to follow-up in the study. LN, lymph node; US, ultrasonography.

Overall, 854 patients were registered. A total of 176 patients were excluded because their cancer primary focus and/or LN metastasis focus had been resected, 40 patients were excluded due to inflammatory breast cancer, and 12 patients were excluded because their necessary clinicopathological information was absent. Ultimately, 228 patients were excluded, and 626 patients were included. Moreover, 14 patients were lost to follow-up because they did not undergo surgical treatment in West China Hospital, and two patients were lost to follow-up due to refusal to undergo preoperative US in West China Hospital. Finally, 16 patients were lost to follow-up, and the remaining 610 patients were included for analysis, which is illustrated in **Figure 1**.

Of these 610 patients, 11 (1.8%) patients had unknown HER2 status, and one patient had unknown Ki-67 status. These missing data were not indispensable for model construction, so these patients were retained without handling their missing data. The mean age at diagnosis of the 610 patients was 50.9 ± 10.7 years (range, 25–86 years). As confirmed by postoperative histopathologic examination, 294 (48.2%) patients had ALNM, while the other 316 (51.8%) patients had negative ALN. In addition, 72 (11.8%) patients underwent NAT. More than half of the patients (55.6%) had tumors of the luminal B subtype, and most patients (94.8%) had ductal carcinoma. In addition, 84.4% of patients had LNs with a short axis <10 mm. The comparison of the clinicopathological and US features of patients between the training set (427 patients) and the test set (183 patients) is shown in **Table 1**. The distribution of variables between the two sets was basically consistent, with a slight difference in the tumor location (**Table 1**).

**Table 1:** Clinicopathological and US features of breast cancer patients in the training and test cohorts.

Features	Total (n = 610) N (%)	Training cohorts (n = 427) N (%)	Test cohorts (n = 183) N (%)	p-Value
ALN status				0.502
Non-ALNM	316 (51.8)	225 (52.7)	91 (49.7)	
ALNM	294 (48.2)	202 (47.3)	92 (50.3)	
Neoadjuvant therapy				0.913
No	538 (88.2)	377 (88.3)	161 (88.0)	
Yes	72 (11.8)	50 (11.7)	22 (12.0)	
Age at diagnosis (years)				0.066
≤50	298 (48.9)	219 (51.3)	79 (43.2)	
>50	312 (51.1)	208 (48.7)	104 (56.8)	
BMI (kg/m^2^)				0.774
<18.5	29 (4.8)	18 (4.2)	11 (6.0)	
≥18.5 and <24	367 (60.2)	259 (60.7)	108 (59.0)	
≥24 and <28	171 (28.0)	120 (28.1)	51 (27.9)	
≥28	43 (7.0)	30 (7.0)	13 (7.1)	
Age at menarche (years)				0.139
≤12	75 (12.3)	58 (13.6)	17 (9.3)	
>12	535 (87.7)	369 (86.4)	166 (90.7)	
The number of pregnancies				0.475
0	33 (5.4)	23 (5.4)	10 (5.5)	
1–2	424 (69.5)	293 (68.6)	131 (71.6)	
≥3	153 (25.1)	111 (26.0)	42 (23.0)	
The number of deliveries				0.507
0	37 (6.1)	25 (5.9)	12 (6.6)	
1–2	548 (89.8)	383 (89.7)	165 (90.2)	
≥3	25 (4.1)	19 (4.4)	6 (3.3)	
Menopause status at diagnosis				0.197
No	291 (47.7)	211 (49.4)	80 (43.7)	
Yes	319 (52.3)	216 (50.6)	103 (56.3)	
ER				0.518
Negative	192 (31.5)	131 (30.7)	61 (33.3)	
Positive	418 (68.5)	296 (69.3)	122 (66.7)	
PR				0.137
Negative	210 (34.4)	139 (32.6)	71 (38.8)	
Positive	400 (65.6)	288 (67.4)	112 (61.2)	
HER2				0.971
Negative	428 (70.2)	301 (70.5)	127 (69.4)	
Positive	171 (28.0)	120 (28.1)	51 (27.9)	
Unknown	11 (1.8)	6 (1.4)	5 (2.7)	
Ki-67				0.333
<14%	160 (26.2)	117 (27.4)	43 (23.5)	
≥14%	449 (73.6)	310 (72.6)	139 (76.0)	
Unknown	1 (0.2)	0 (0.0)	1 (0.5)	
Molecular subtypes				0.206
Luminal A	100 (16.4)	78 (18.3)	22 (12.0)	
Luminal B	339 (55.6)	234 (54.8)	105 (57.4)	
HER2-enriched	72 (11.8)	51 (11.9)	21 (11.5)	
Triple-negative	99 (16.2)	64 (15.0)	35 (19.1)	
Histologic types				0.498
Ductal	578 (94.8)	404 (94.6)	174 (95.1)	
Lobular	16 (2.6)	13 (3.0)	3 (1.6)	
Others	16 (2.6)	10 (2.3)	6 (3.3)	
Tumor size (cm)				0.892
≤2	274 (44.9)	193 (45.2)	81 (44.3)	
>2 and ≤5	317 (52.0)	220 (51.5)	97 (53.0)	
>5	19 (3.1)	14 (3.3)	5 (2.7)	
Tumor location				0.006*
Upper lateral quadrant	301 (49.3)	195 (45.7)	106 (57.9)	
Others	309 (50.7)	232 (54.3)	77 (42.1)	
Distance to the nipple				0.513
≤2 cm	311 (51.0)	214 (50.1)	97 (53.0)	
>2 cm	299 (49.0)	213 (49.9)	86 (47.0)	
Blood flow signals of the tumor				0.831
Poor	276 (45.2)	192 (45.0)	84 (45.9)	
Abundant	334 (54.8)	235 (55.0)	99 (54.1)	
Infiltration of subcutaneous layer				0.355
No	373 (61.1)	256 (60.0)	117 (63.9)	
Yes	237 (38.9)	171 (40.0)	66 (36.1)	
Infiltration of retromammary space				0.985
No	423 (69.3)	296 (69.3)	127 (69.4)	
Yes	187 (30.7)	131 (30.7)	56 (30.6)	
LN long axis (mm)				0.750
<20	475 (77.9)	331 (77.5)	144 (78.7)	
≥20	135 (22.1)	96 (22.5)	39 (21.3)	
LN short axis (mm)				0.370
<10	515 (84.4)	364 (85.2)	151 (82.5)	
≥10 and <15	69 (11.3)	47 (11.0)	22 (12.0)	
≥15	26 (4.3)	16 (3.7)	10 (5.5)	
LN L/S axis ratio				0.846
>2	430 (70.5)	300 (70.3)	130 (71.0)	
≤2	180 (29.5)	127 (29.7)	53 (29.0)	
Blood flow signal types of LN				0.707
None	376 (61.6)	269 (63.0)	107 (58.5)	
Portal	177 (29.0)	121 (28.3)	56 (30.6)	
Peripheral	39 (6.4)	25 (5.9)	14 (7.7)	
Mixed	18 (3.0)	12 (2.8)	6 (3.3)	
Blood flow signal grades of LN				0.440
0	376 (61.6)	269 (63.0)	107 (58.5)	
1	151 (24.8)	97 (22.7)	54 (29.5)	
2	51 (8.4)	41 (9.6)	10 (5.5)	
3	32 (5.2)	20 (4.7)	12 (6.6)	
LN margin				0.420
Distinct	463 (75.9)	328 (76.8)	135 (73.8)	
Obscure	147 (24.1)	99 (23.2)	48 (26.2)	
LN corticomedullary demarcation				0.478
Distinct	455 (74.6)	315 (73.8)	140 (76.5)	
Obscure	155 (25.4)	112 (26.2)	43 (23.5)	
LN cortical thickness				0.911
≤2 mm	398 (65.2)	278 (65.1)	120 (65.6)	
>2 mm	212 (34.8)	149 (34.9)	63 (34.4)	
LN cortical thickness evenness				0.615
Even	401 (65.7)	278 (65.1)	123 (67.2)	
Uneven	209 (34.3)	149 (34.9)	60 (32.8)	

US, ultrasonography; LN, lymph node; ALN, axillary lymph node; ALNM, axillary lymph node metastasis; BMI, body mass index; ER, estrogen receptor; PR, progesterone receptor; HER2, human epidermal growth factor receptor 2; L/S, long/short.

* Statistically significant.

The clinicopathological and US features of patients with or without ALNM in the training and test cohorts are summarized in **Table 2**. In the training cohort, 202 (47.3%) patients had ALNM, and the remaining 225 (52.7%) patients had negative ALN. In the test cohort, 92 (50.3%) patients had ALNM, and the remaining 91 (49.7%) patients had negative ALN.

**Table 2:** Clinicopathological and US features of breast cancer patients with or without ALNM in the training and test cohorts.

Features	Training cohorts (n = 427)		Test cohorts (n = 183)	
	ALNM (n = 202) N (%)	Non-ALNM (n = 225) N (%)	ALNM (n = 92) N (%)	Non-ALNM (n = 91) N (%)
Neoadjuvant therapy				
No	176 (87.1)	201 (89.3)	79 (85.9)	82 (90.1)
Yes	26 (12.9)	24 (10.7)	13 (14.1)	9 (9.9)
Age at diagnosis (years)				
≤50	97 (48.0)	122 (54.2)	41 (44.6)	38 (41.8)
>50	105 (52.0)	103 (45.8)	51 (55.4)	53 (58.2)
BMI (kg/m^2^)				
<18.5	7 (3.5)	11 (4.9)	4 (4.3)	7 (7.7)
≥18.5 and <24	115 (56.9)	144 (64.0)	54 (58.7)	54 (59.3)
≥24 and <28	63 (31.2)	57 (25.3)	28 (30.4)	23 (25.3)
≥28	17 (8.4)	13 (5.8)	6 (6.5)	7 (7.7)
Age at menarche (years)				
≤12	25 (12.4)	33 (14.7)	8 (8.7)	9 (9.9)
>12	177 (87.6)	192 (85.3)	84 (91.3)	82 (90.1)
The number of pregnancies				
0	7 (3.5)	16 (7.1)	0 (0.0)	10 (11.0)
1–2	138 (68.3)	155 (68.9)	64 (69.6)	67 (73.6)
≥3	57 (28.2)	54 (24.0)	28 (30.4)	14 (15.4)
The number of deliveries				
0	9 (4.5)	16 (7.1)	1 (1.1)	11 (12.1)
1–2	183 (90.6)	200 (88.9)	86 (93.5)	79 (86.8)
≥3	10 (5.0)	9 (4.0)	5 (5.4)	1 (1.1)
Menopause status at diagnosis				
No	95 (47.0)	116 (51.6)	41 (44.6)	39 (42.9)
Yes	107 (53.0)	109 (48.4)	51 (55.4)	52 (57.1)
ER				
Negative	51 (25.2)	80 (35.6)	27 (29.3)	34 (37.4)
Positive	151 (74.8)	145 (64.4)	65 (70.7)	57 (62.6)
PR				
Negative	57 (28.2)	82 (36.4)	29 (31.5)	42 (46.2)
Positive	145 (71.8)	143 (63.6)	63 (68.5)	49 (53.8)
HER2				
Negative	145 (71.8)	156 (69.3)	65 (70.7)	62 (68.1)
Positive	56 (27.7)	64 (28.4)	26 (28.3)	25 (27.5)
Unknown	1 (0.5)	5 (2.2)	1 (1.1)	4 (4.4)
Ki-67				
<14%	48 (23.8)	69 (30.7)	20 (21.7)	23 (25.3)
≥14%	154 (76.2)	156 (69.3)	72 (78.3)	67 (73.6)
Unknown	0 (0.0)	0 (0.0)	0 (0.0)	1 (1.1)
Molecular subtypes				
Luminal A	38 (18.8)	40 (17.8)	11 (12.0)	11 (12.1)
Luminal B	122 (60.4)	112 (49.8)	58 (63.0)	47 (51.6)
HER2-enriched	21 (10.4)	30 (13.3)	8 (8.7)	13 (14.3)
Triple-negative	21 (10.4)	43 (19.1)	15 (16.3)	20 (22.0)
Histologic types				
Ductal	198 (98.0)	206 (91.6)	90 (97.8)	84 (92.3)
Lobular	3 (1.5)	10 (4.4)	2 (2.2)	1 (1.1)
Others	1 (0.5)	9 (4.0)	0 (0.0)	6 (6.6)
Tumor size (cm)				
≤2	70 (34.7)	123 (54.7)	33 (35.9)	48 (52.7)
>2 and ≤5	120 (59.4)	100 (44.4)	55 (59.8)	42 (46.2)
>5	12 (5.9)	2 (0.9)	4 (4.3)	1 (1.1)
Tumor location				
Upper lateral quadrant	98 (48.5)	97 (43.1)	58 (63.0)	48 (52.7)
Others	104 (51.5)	128 (56.9)	34 (37.0)	43 (47.3)
Distance to the nipple				
≤2 cm	102 (50.5)	112 (49.8)	52 (56.5)	45 (49.5)
>2 cm	100 (49.5)	113 (50.2)	40 (43.5)	46 (50.5)
Blood flow signals of the tumor				
Poor	69 (34.2)	123 (54.7)	38 (41.3)	46 (50.5)
Abundant	133 (65.8)	102 (45.3)	54 (58.7)	45 (49.5)
Infiltration of subcutaneous layer				
No	87 (43.1)	169 (75.1)	55 (59.8)	62 (68.1)
Yes	115 (56.9)	56 (24.9)	37 (40.2)	29 (31.9)
Infiltration of retromammary space				
No	109 (54.0)	187 (83.1)	53 (57.6)	74 (81.3)
Yes	93 (46.0)	38 (16.9)	39 (42.4)	17 (18.7)
LN long axis (mm)				
<20	141 (69.8)	190 (84.4)	62 (67.4)	82 (90.1)
≥20	61 (30.2)	35 (15.6)	30 (32.6)	9 (9.9)
LN short axis (mm)				
<10	149 (73.8)	215 (95.6)	64 (69.6)	87 (95.6)
≥10 and <15	38 (18.8)	9 (4.0)	18 (19.6)	4 (4.4)
≥15	15 (7.4)	1 (0.4)	10 (10.9)	0 (0.0)
LN L/S axis ratio				
>2	112 (55.4)	188 (83.6)	49 (53.3)	81 (89.0)
≤2	90 (44.6)	37 (16.4)	43 (46.7)	10 (11.0)
Blood flow signal types of LN				
None	114 (56.4)	155 (68.9)	45 (48.9)	62 (68.1)
Portal	53 (26.2)	68 (30.2)	27 (29.3)	29 (31.9)
Peripheral	24 (11.9)	1 (0.4)	14 (15.2)	0 (0.0)
Mixed	11 (5.4)	1 (0.4)	6 (6.5)	0 (0.0)
Blood flow signal grades of LN				
0	114 (56.4)	155 (68.9)	45 (48.9)	62 (68.1)
1	51 (25.2)	46 (20.4)	31 (33.7)	23 (25.3)
2	23 (11.4)	18 (8.0)	7 (7.6)	3 (3.3)
3	14 (6.9)	6 (2.7)	9 (9.8)	3 (3.3)
LN margin				
Distinct	128 (63.4)	200 (88.9)	56 (60.9)	79 (86.8)
Obscure	74 (36.6)	25 (11.1)	36 (39.1)	12 (13.2)
LN corticomedullary demarcation				
Distinct	105 (52.0)	210 (93.3)	51 (55.4)	89 (97.8)
Obscure	97 (48.0)	15 (6.7)	41 (44.6)	2 (2.2)
LN cortical thickness				
≤2 mm	102 (50.5)	176 (78.2)	52 (56.5)	68 (74.7)
>2 mm	100 (49.5)	49 (21.8)	40 (43.5)	23 (25.3)
LN cortical thickness evenness				
Even	102 (50.5)	176 (78.2)	50 (54.3)	73 (80.2)
Uneven	100 (49.5)	49 (21.8)	42 (45.7)	18 (19.8)

US, ultrasonography; LN, lymph node; ALNM, axillary lymph node metastasis; BMI, body mass index; ER, estrogen receptor; PR, progesterone receptor; HER2, human epidermal growth factor receptor 2; L/S, long/short.

### Univariate analysis of ALNM

Univariate binary logistic regression analysis was conducted in the training set (**Table 3**). According to preoperative histopathologic biopsy, ER-positive, luminal A/B subtype, or ductal carcinoma was associated with ALNM. In addition, larger tumor size, abundant blood flow signals of the tumor, and tumor infiltration of the subcutaneous layer or retromammary space reported in preoperative US of breasts were also associated with ALNM. Moreover, according to preoperative US for ALN, longer LN long axis or short axis, smaller LN L/S axis ratio, peripheral or mixed blood flow signal type of LN, grade 3 blood flow signal of LN, obscure LN margin or corticomedullary demarcation, and thicker or more uneven LN cortex were also associated with ALNM.

**Table 3:** Univariate and multivariate binary logistic regression analyses of clinicopathological and US features associated with ALNM.

Features	Univariate analysis		Multivariate analysis	
	p-Value	OR (95% CI)	Adjusted p-value	Adjusted OR (95% CI)
Age at diagnosis (>50 vs. ≤50 years)	0.201	1.282 (0.876–1.876)		
BMI (kg/m^2^)	0.294			
≥18.5 and <24 vs. <18.5	0.649	1.255 (0.472–3.340)		
≥24 and <28 vs. <18.5	0.286	1.737 (0.631–4.783)		
≥28 vs. <18.5	0.236	2.055 (0.624–6.764)		
Age at menarche (>12 vs. ≤12 years)	0.491	1.217 (0.696–2.127)		
The number of pregnancies	0.197			
1–2 vs. 0	0.129	2.035 (0.813–5.093)		
≥3 vs. 0	0.073	2.413 (0.921–6.320)		
The number of deliveries	0.470			
1–2 vs. 0	0.257	1.627 (0.702–3.771)		
≥3 vs. 0	0.272	1.975 (0.586–6.662)		
Menopause status at diagnosis (yes vs. no)	0.351	1.199 (0.819–1.753)		
ER (positive vs. negative)	0.022*	1.634 (1.075–2.483)	0.269	
PR (positive vs. negative)	0.071	1.459 (0.969–2.197)		
HER2 (positive vs. negative)	0.780	0.941 (0.616–1.438)		
Ki-67 (≥14% vs. <14%)	0.111	1.419 (0.923–2.182)		
Molecular subtypes	0.041*		0.015*	
Luminal A vs. TN	0.057	1.945 (0.980–3.859)	0.003	3.611 (1.545–8.442)
Luminal B vs. TN	0.007	2.230 (1.247–3.989)	0.026	2.288 (1.104–4.740)
HER2-enriched vs. TN	0.356	1.433 (0.668–3.076)	0.626	1.290 (0.463–3.592)
Histologic types	0.029*		0.069	
Lobular vs. ductal	0.080	0.312 (0.085–1.151)		
Others vs. ductal	0.042	0.116 (0.015–0.921)		
Tumor size (cm)	<0.001*		0.338	
>2 and ≤5 vs. ≤2	<0.001	2.109 (1.420–3.132)		
>5 vs. ≤2	0.002	10.543 (2.293–48.467)		
Tumor location (others vs. ULQ)	0.263	0.804 (0.549–1.178)		
Distance to the nipple (>2 vs. ≤2 cm)	0.882	0.972 (0.665–1.421)		
Blood flow signals of the tumor (abundant vs. poor)	<0.001*	2.324 (1.571–3.439)	0.121	
Infiltration of subcutaneous layer (yes vs. no)	<0.001*	3.989 (2.645–6.017)	<0.001*	2.755 (1.627–4.663)
Infiltration of retromammary space (yes vs. no)	<0.001*	4.199 (2.690–6.553)	0.001*	2.534 (1.443–4.452)
LN long axis (≥20 vs. <20 mm)	<0.001*	2.349 (1.469–3.755)	0.158	
LN short axis (mm)	<0.001*		0.007*	
≥10 and <15 vs. <10	<0.001	6.092 (2.861–12.976)	0.010	3.354 (1.329–8.461)
≥15 vs. <10	0.003	21.644 (2.829–165.627)	0.049	9.696 (1.006–93.416)
LN L/S axis ratio (≤2 vs. >2)	<0.001*	4.083 (2.607–6.394)	0.019*	2.003 (1.121–3.577)
Blood flow signal types of LN	<0.001*		0.113	
Portal vs. none	0.793	1.060 (0.687–1.634)		
Peripheral vs. none	0.001	32.632 (4.351–244.747)		
Mixed vs. none	0.010	14.956 (1.904–117.504)		
Blood flow signal grades of LN	0.032*		0.550	
1 vs. 0	0.084	1.507 (0.946–2.403)		
2 vs. 0	0.102	1.737 (0.896–3.370)		
3 vs. 0	0.022	3.173 (1.183–8.508)		
LN margin (obscure vs. distinct)	<0.001*	4.625 (2.792–7.662)	0.350	
LN corticomedullary demarcation (obscure vs. distinct)	<0.001*	12.933 (7.154–23.381)	<0.001*	8.405 (4.186–16.876)
LN cortical thickness (>2 vs. ≤2 mm)	<0.001*	3.521 (2.314–5.359)	0.768	
LN cortical thickness evenness (uneven vs. even)	<0.001*	3.521 (2.314–5.359)	0.024*	1.842 (1.085–3.129)

US, ultrasonography; LN, lymph node; ALNM, axillary lymph node metastasis; OR, odds ratio; CI, confidence interval; BMI, body mass index; ER, estrogen receptor; PR, progesterone receptor; HER2, human epidermal growth factor receptor 2; TN, triple-negative; ULQ, upper lateral quadrant; L/S, long/short.

* Statistically significant.

### Multivariate analysis of ALNM

As shown in **Table 3**, multivariate binary regression analysis revealed that molecular subtypes, tumor infiltration of the subcutaneous layer, tumor infiltration of the retromammary space, LN short axis, LN L/S axis ratio, LN corticomedullary demarcation, and LN cortical thickness evenness were independent predictors of ALNM. Compared with triple-negative (TN) subtypes, luminal A (adjusted OR [95% CI], 3.611 [1.545–8.442], p = 0.003) and B (adjusted OR [95% CI], 2.288 [1.104–4.740], p = 0.026) subtypes were both more prone to ALNM. In addition, tumor infiltration of the subcutaneous layer (adjusted OR [95% CI], 2.755 [1.627–4.663], p < 0.001) and the retromammary space (adjusted OR [95% CI], 2.534 [1.443–4.452], p = 0.001) were both risk factors of ALNM. Moreover, the risk of metastasis greatly increased when the LN short axis was too long. Compared with short axis <10 mm, ≥10 and <15 mm indicated that ALNM risk was more than three times (adjusted OR [95% CI], 3.354 [1.329–8.461], p = 0.010), while short axis ≥15 mm indicated that ALNM risk was nearly 10 times (adjusted OR [95% CI], 9.696 [1.006–93.416], p = 0.049). LNs with L/S axis ratio ≤2 had a higher risk of metastasis than LNs with L/S axis ratio >2 (adjusted OR [95% CI], 2.003 [1.121–3.577], p = 0.019). In addition, LNs with obscure corticomedullary demarcation (adjusted OR [95% CI], 8.405 [4.186–16.876)], p < 0.001) had a great risk of metastasis, and uneven cortex (adjusted OR [95% CI], 1.842 [1.085–3.129], p = 0.024) also increased the metastasis risk of LN. According to the multicollinearity test, there was no collinearity among these independent predictors (tolerance > 0.2 and VIF < 5).

### Nomogram development and test

**Figure 2:**
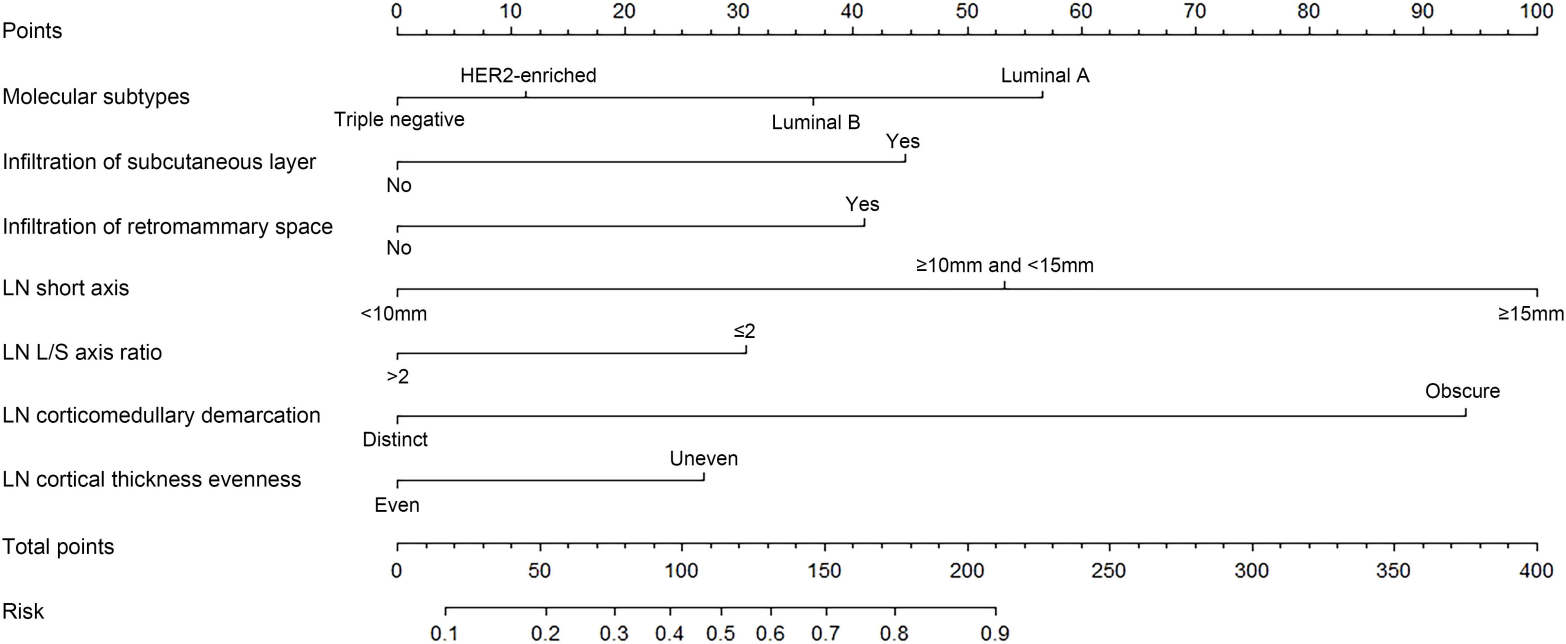
A nomogram to predict axillary lymph node (LN) metastasis (ALNM) preoperatively in patients with breast cancer. Molecular subtypes, tumor infiltration of subcutaneous layer, tumor infiltration of retromammary space, LN short axis, LN long/short (L/S) axis ratio, LN corticomedullary demarcation, and LN cortical thickness evenness were finally selected to develop the model.

A binary logistic regression model to predict the probability of ALNM was developed based on the abovementioned independent predictors and presented as a nomogram (**Figure 2**). The Hosmer–Lemeshow goodness-of-fit test suggested that the model fit well (p = 0.770).

**Figure 3:**
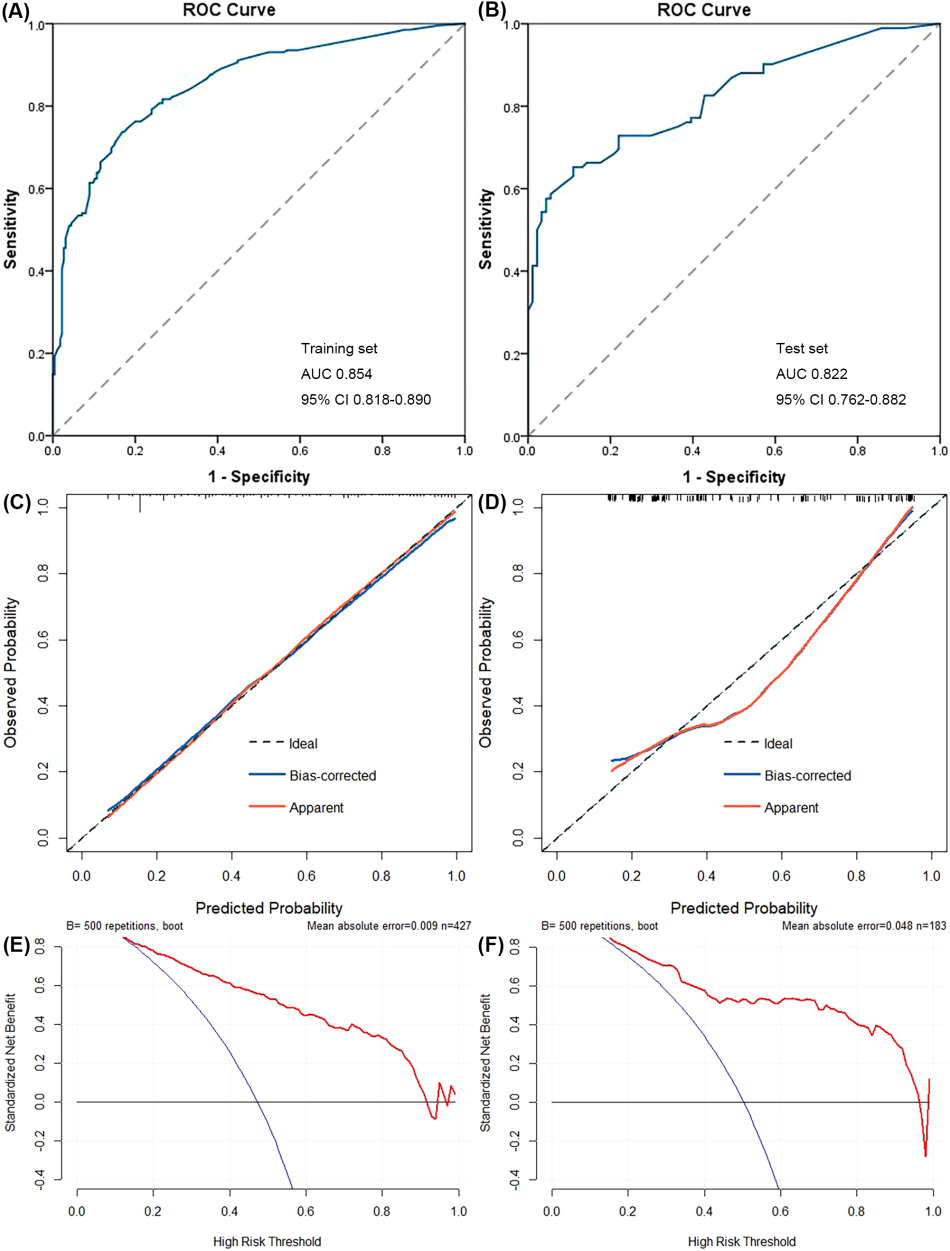
Performance of the nomogram was evaluated using the receiver operating characteristic (ROC) curves **(A, B)**, the calibration curves **(C, D)**, and the decision curve analysis (DCA) **(E, F)**. The area under ROC curve (AUC) of the model was 0.854 (95% CI, 0.818–0.890) and 0.822 (95% CI, 0.762–0.882) for the training set **(A)** and the test set **(B)**, respectively. After 500 resampling, the mean absolute error of the model was 0.009 and 0.048 for the training set **(C)** and the test set **(D)**, respectively. The model could acquire net benefit when the risk threshold was located at 0.15–0.9 and 0.15–0.95 for the training set **(E)** and the test set **(F)**, respectively.

According to further evaluation, the model had good discrimination ability with AUCs of 0.854 (95% CI, 0.818–0.890) and 0.822 (95% CI, 0.762–0.882) for the training set and the test set, respectively (**Figures 3A, B**). Moreover, the calibration curves indicated good agreement between predicted and observed probabilities, with the mean absolute error of 0.009 and 0.048 (500 repetitions) for the training set and the test set, respectively (**Figures 3C, D**). In the test set, when the observed probability was in the middle range (approximately 0.3–0.85), the model slightly overestimated the risk; however, when the observed probability was low or high (roughly <0.3 or >0.85), the model slightly underestimated the risk. DCA curves showed that the model could acquire net benefit with a threshold range of roughly 0.15–0.9 and 0.15–0.95 for the training group and the test group, respectively (**Figures 3E, F**). In particular, the model acquired greater net benefit (≥0.4) when the threshold range was approximately 0.15–0.65 and 0.15–0.8 for the training group and test group, respectively. Therefore, it was demonstrated that the model was of great benefit to guide clinical decisions for predicting ALNM.

As shown in **Table 4**, with a risk threshold value of 0.5, the sensitivity, specificity, and accuracy of the model were 71.3%, 84.9%, and 78.5%, respectively, for the training set and 65.2%, 87.9%, and 76.5%, respectively, for the test set. Meanwhile, the detection rates were 41.7% and 38.8% in the training set and the test set, respectively, and these patients were identified to have ALNM and were likely to undergo further invasive examinations. In addition, the performance at different threshold values was presented to provide references for threshold value selection in situations with different requirements. For example, with a threshold value of 0.231, the sensitivity, specificity, and accuracy were 90.6%, 55,1%, and 71.9%, respectively, for the training set and 82.6%, 57.1%, and 69.9%, respectively, for the test set; the detection rates were 66.5% and 62.8% in the training set and the test set, respectively.

**Table 4:** Performance of the logistic regression model for ALNM prediction in the training and test cohorts when the threshold value varied.

Cohorts	Threshold value	Sensitivity (%)	Specificity (%)	Accuracy (%)	PPV (%)	NPV (%)	Detection rate (%)	FNR (%)
Training cohort	0.080	99.5	6.7	50.6	48.9	93.8	96.3	0.5
	0.140	98.0	16.9	55.3	51.4	90.5	90.2	2.0
	0.231	90.6	55.1	71.9	64.4	86.7	66.5	9.4
	0.315	86.6	62.7	74.0	67.6	83.9	60.7	13.4
	0.408	80.2	74.7	77.3	74.0	80.8	51.3	19.8
	0.5	71.3	84.9	78.5	80.9	76.7	41.7	28.7
Test cohort	0.080	98.9	8.8	54.1	52.3	88.9	95.1	1.1
	0.140	98.9	14.3	56.8	53.8	92.9	92.3	1.1
	0.231	82.6	57.1	69.9	66.1	76.5	62.8	17.4
	0.315	75.0	63.7	69.4	67.6	71.6	55.7	25.0
	0.408	69.6	78.0	73.8	84.5	71.4	45.9	30.4
	0.5	65.2	87.9	76.5	84.5	71.4	38.8	34.8

ALNM, axillary lymph node metastasis; PPV, positive predictive value; NPV, negative predictive value; FNR, false-negative rate.

## Discussion

In this study, we prospectively included patients with breast cancer and collected preoperative clinicopathological features and US features of tumors and LNs to explore the risk factors of ALNM. The results suggested that molecular subtypes, tumor infiltration of the subcutaneous layer, tumor infiltration of the retromammary space, LN short axis, LN L/S axis ratio, LN corticomedullary demarcation, and LN cortical thickness evenness were independent predictors of ALNM. Then, we developed a logistic regression model based on these predictors and presented it as a nomogram to predict ALNM, displaying a good performance in discrimination ability, calibration ability, and net benefit for both the training set and test set.

In our predictive model, LN corticomedullary demarcation was one of the most important predictors, showing the highest predictive value, and LN cortical thickness evenness also exhibited a slight predictive value. LNs with obscure corticomedullary demarcation had more than eight times the risk of metastasis than LNs with distinct corticomedullary demarcation, while LNs with uneven cortex had nearly two times the risk than LNs with even cortex. Previous studies also suggested that corticomedullary demarcation (34) and asymmetrical cortex (33) were independent predictors of ALNM. In addition, LN short axis was one of the most important predictors, and the L/S axis ratio also presented some predictive value. Compared with short axis <10 mm, ≥10 and <15 mm indicated that ALNM risk was more than three times, while short axis ≥15 mm indicated that ALNM risk was nearly 10 times. Rounder LNs (L/S axis ratio ≤2) had roughly two times the risk of metastasis than oval LNs (L/S axis ratio >2). Studies have also reported that LN short diameter (29) and LN L/S axis ratio (33) were independent predictors of ALNM. Overall, studies have indicated that both LN size and morphological features were equally important for LN metastasis prediction (39, 40), which was consistent with our results.

Molecular subtypes were also an important independent predictor of ALNM, but the specific relationship is quite controversial. Although some studies have failed to discover the association between the molecular subtypes and the ALN status (41), many studies have revealed that molecular subtypes were associated with ALNM (27, 42–52). Some studies have shown that non-luminal subtypes (TN or HER2-enriched) were associated with a higher risk of ALNM (45, 52) and that the luminal A subtype had a lower risk of ALNM than the other subtypes (27, 45, 52). Other studies have suggested that luminal subtypes (A or B) were more prone to ALNM (43, 44, 47, 51), which was consistent with our study. In our study, adjusted by multivariate analysis, tumors of the luminal A subtype had more than three times the risk of ALNM than tumors of TN, while the luminal B subtype had more than two times the risk, and although the luminal B subtype seemed to have a higher risk before adjustment, there was no significant difference between the luminal A and B subtypes. As is known to us, TN is always associated with a worse prognosis, but many studies (including ours) have shown that TN has a lower risk of ALNM (43, 44, 51). Therefore, the poor prognosis of TN may be due to distant spread, rather than regional spread (45, 51). In addition, the luminal A subtype is always considered to have the best prognosis (27), but many studies (including ours) have suggested that it was more prone to ALNM than TN and HER2-enriched (44, 47). The reason may be that the luminal A subtype has positive ER and high expression of PR, and positive ER (29, 53) or PR (34, 54, 55) may mean a higher risk of ALNM according to previous studies. The specific mechanism of the phenomenon has been unknown, but we deduced that tumors of luminal subtypes may prefer lymphatic metastasis, and thus, patients are more likely to benefit from local therapy; also, tumors of non-luminal subtypes may prefer hematogenous metastasis, and thus, distant metastasis may occur earlier (29).

In our study, two variables of the primary tumor were incorporated into the model innovatively: tumor infiltration of the subcutaneous layer and tumor infiltration of the retromammary space. The results indicated that both of them were independent predictors of ALNM, and patients with one of the infiltration patterns above had more than two times the risk of ALNM than patients with neither pattern. A previous study suggested that infiltration of subcutaneous adipose tissue (ISAT) was an independent predictor of ALNM, and tumors with ISAT were 2.72 times more likely to develop ALNM than those without ISAT (56), which was consistent with our study. Lymphatic capillaries are densely distributed at subcutaneous adipose tissue (57, 58), which may make tumors with infiltration of the subcutaneous layer more prone to ALNM. In addition, another study also showed that infiltration of the retromammary space was associated with ALNM, but the specific mechanism has been unknown (59). The deep lymphatics of breasts drain through the retromammary space (60), which may be an underlying mechanism.

In recent years, increasing prediction models based on imaging examinations, particularly US, have been developed to evaluate the ALN status of breast cancer preoperatively. However, the features incorporated in these studies were inadequate. Some studies incorporated US features of LNs only (29, 32, 34), and another study incorporated US features of primary tumors only (27). For a study incorporating the US features of both tumors and LNs, the clinicopathological characteristics were from surgical specimens, which also limited the preoperative application of the model (33). We prospectively comprehensively incorporated preoperative US features of tumors and LNs, as well as acquired clinicopathological characteristics from preoperative biopsy, and developed a nomogram to predict ALN status preoperatively, successfully resolving the questions raised above.

The American College of Surgeons Oncology Group Z0011 (ACOSOGZ0011) randomized clinical trial suggested no benefit from ALND for patients with ≤2 SLN metastasis and receiving standard therapy (61). For more than 20 years, SLNB has been the standard for ALN staging in early breast cancer to identify patients benefiting from ALND, which represented a milestone in surgical de-escalation (15). The SOUND trial showed that omitting axillary surgery was non-inferior to SLNB in patients with tumors ≤2 cm and negative ALN US (15), which may become another milestone in surgical de-escalation. Therefore, preoperative ALN evaluation is critical for identifying patients who can safely omit axillary surgery (29). To be exact, our study provided an important reference for the accurate prediction of preoperative ALN status based on non-invasive techniques and preliminary evidence for the precise selection of patients who can omit SLNB safely.

Our study has some advantages. First, the patients were prospectively included, and the features were prospectively collected. Second, the US features of the primary tumors and LNs were incorporated relatively comprehensively, and clinicopathological characteristics were all acquired from biopsy preoperatively, which was beneficial for the preoperative sufficient evaluation of ALN status and corresponded more with determination scenarios of SLNB omission. Third, patients who underwent NAT were included, which broadened the application of the nomogram. At last, the model was evaluated relatively comprehensively from the three dimensions: discrimination, calibration, and net benefits. Nevertheless, our study also has some limitations. First, our study was a single-center study, and it needs to be further tested in external test cohorts from other institutions to evaluate its predictive ability and generalizability. Second, there were still slight differences between the training and test sets, even if the division was random. Third, US features depend on ultrasound specialists’ judgments, which are subjective and inevitably biased. At last, manual feature extraction to construct logistic regression models is simpler but less abundant, compared with radiomics feature extraction by artificial intelligence to develop machine learning or deep learning models.

## Conclusion

In conclusion, molecular subtypes, tumor infiltration of the subcutaneous layer, tumor infiltration of the retromammary space, LN short axis, LN L/S axis ratio, LN corticomedullary demarcation, and LN cortical thickness evenness were independent predictors of ALNM in breast cancer. Based on these independent predictors, we developed a logistic regression model and presented it as a nomogram to predict ALNM, which displayed a good performance in discrimination ability, calibration ability, and net benefits for both the training set and test set, and it could assist clinical decisions.

## Data Availability

The raw data supporting the conclusions of this article will be made available by the authors, without undue reservation.
